# Whole-genome sequencing of carbapenem-resistant Enterobacterales isolates and evaluation of hospital-acquired infections

**DOI:** 10.1017/ash.2023.335

**Published:** 2023-09-29

**Authors:** Leama Ajaka, Shandra Day, Christina Liscynesky, Nora Colburn, Christine Sun, Michael Sovic, Preeti Pancholi, Joan-Miquel Balada-Llasat, Heather Smith, Shashanka Murthy

## Abstract

**Background:** Multidrug-resistant organisms (MDROs) are increasingly implicated in nosocomial outbreaks worldwide. We evaluated whole-genome sequencing (WGS) as an adjunctive epidemiological tool to identify infection clusters and MDRO transmission in the healthcare setting. **Methods:** Clinical isolates of carbapenem-resistant Enterobacterales (CRE) from July 1, 2021, to June 30, 2022, underwent Illumina WGS. Assembled genomes were taxonomically classified with GTDB-Tk software and were typed using multilocus sequence typing (MLST). Average nucleotide identity (ANI) was calculated between genomes. Numbers of differences among core single-nucleotide polymorphisms (SNPs) were calculated for pairs within taxonomic groups, and the data were evaluated in the context of patient dates and locations of care obtained from the electronic medical record. **Results:** In total, 39 CRE isolates underwent WGS (Fig. 1). *Klebsiella pneumoniae* represented the largest number of isolates (n = 18). Using MLST, 2 distinct groups of *K. pneumoniae* were identified (ST307 and ST258) with 5 and 4 isolates, respectively (Fig. 2). Within ST307, SNP differences ranged between 8 and 115. 3 isolates (CRE8, CRE10, and CRE12) were collected within 4 weeks of each other and had ≤26 pairwise SNP differences. Notably, CRE8 and CRE10 were located on the same unit at the same time and used the same MRI scanner on the same day. CRE35 had >95 SNP differences and was admitted 8 months prior to others in ST307 but had surgery in the same OR as CRE8. Within ST258, pairwise comparison of samples revealed 139–588 SNP differences. CRE21, CRE31, and CRE33 had SNP differences of ≤150. These patients were in the same hospital room (CRE33 and CRE21) and unit (CRE31 and CRE33), but they did not overlap temporally. CRE37 had >580 SNP differences, with no overlap in hospitalization dates or locations with other patients. **Conclusions:** Two closely related *K. pneumoniae* isolate populations were identified using WGS. Strong temporal and spatial commonalities were identified among isolates with few SNP differences. Isolate pairs with intermediate SNP differences shared spatial commonalities, suggesting possible indirect transmission between patients. No common exposures were identified for pairs with large numbers of SNP differences. WGS is an evolving tool to detect outbreak clonal populations of MDRO not identified through traditional epidemiologic techniques. WGS can provide insight into transmission patterns and the role of environmental contamination in propagating these nosocomial infections. More studies are needed to define the role and clinical significance of isolates with intermediate SNP differences in transmission of these pathogens between hosts and the healthcare environment.

**Figure:**
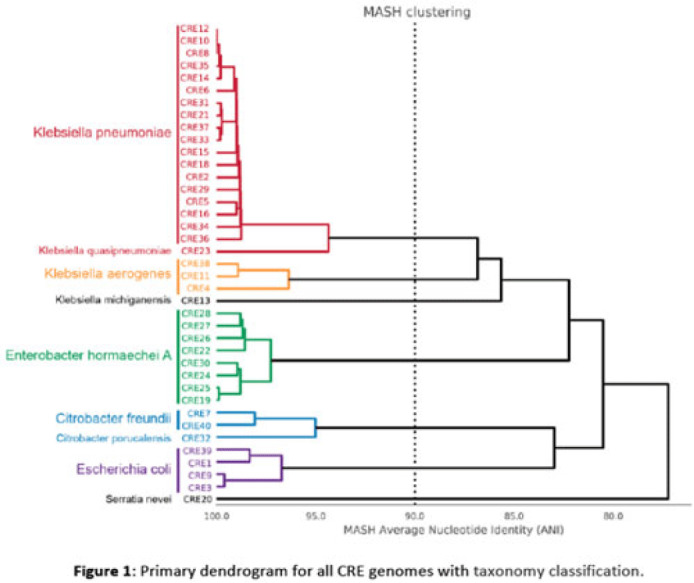

**Figure:**
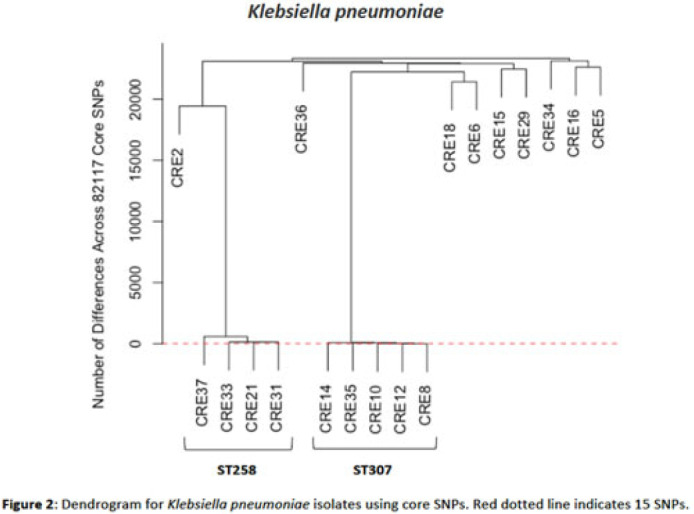

**Disclosures:** None

